# Rough sets: past, present, and future

**DOI:** 10.1007/s11047-018-9700-3

**Published:** 2018-07-25

**Authors:** Andrzej Skowron, Soma Dutta

**Affiliations:** 1 Faculty of Mathematics, Informatics and Mechanics, University of Warsaw, Banacha 2, 02-097 Warsaw, Poland; 2 Systems Research Institute, Polish Academy of Sciences, Newelska 6, 01-447 Warsaw, Poland; 3 Vistula University, Stokłosy 3, 02-787 Warsaw, Poland; 4 Department of Mathematics and Computer Science, University of Warmia and Mazury, Sloneczna str. 54, 10–710 Olsztyn, Poland

**Keywords:** Rough set, Granular computing, (Approximate) Boolean reasoning, Interaction, Adaptive judgment, Complex adaptive system, Natural computing

## Abstract

Introduction of rough sets by Professor Zdzisław Pawlak has completed 35 years. The theory has already attracted the attention of many researchers and practitioners, who have contributed essentially to its development, from all over the world. The methods, developed based on rough set theory alone or in combination with other approaches, found applications in many areas. In this article, we outline some selected past and present research directions of rough sets. In particular, we emphasize the importance of searching strategies for relevant approximation spaces as the basic tools in achieving computational building blocks (granules or patterns) required for approximation of complex vague concepts. We also discuss new challenges related to problem solving by intelligent systems (IS) or complex adaptive systems (CAS). The concern is to control problems using interactive granular computing, an extension of the rough set approach, for effective realization of computations realized in IS or CAS. These challenges are important for the development of natural computing too.

## Introduction

The rough set approach was proposed by Pawlak (1982, 1991) as a tool for dealing with imperfect knowledge, in particular with vague concepts. Rough set theory has gained interest of many researchers and practitioners from all over the world.

The rough set approach is of fundamental importance in artificial intelligence and cognitive sciences, especially in machine learning, data mining and knowledge discovery, pattern recognition, decision support systems, expert systems, intelligent systems, multiagent systems, (complex) adaptive systems, autonomous systems, cognitive systems, conflict analysis, risk management systems.

Many methods based on rough sets have wide applications in many real life projects, e.g., acoustics, bioinformatics, business and finance, chemistry, computer engineering and electrical engineering (including data compression, control, digital image processing, digital signal processing, parallel and distributed computer systems, power systems, sensor fusion, fractal engineering), decision analysis and systems, economics, environmental studies, digital image processing, informatics, medicine, molecular biology, musicology, neurology, robotics, social science, software engineering, spatial visualization, Web engineering, and Web mining.

Rough sets have established relationships with many other approaches such as fuzzy set theory, granular computing, evidence theory, formal concept analysis, (approximate) Boolean reasoning, multicriteria decision analysis, statistical methods, decision theory, matroids. Despite the overlap with many other theories, rough set theory may be considered as an independent discipline on its own right. There are reports on many hybrid methods obtained by combining rough sets with other approaches such as soft computing (fuzzy sets, neural networks, genetic algorithms), statistics, natural computing, mereology, principal component analysis, singular value decomposition or support vector machines.

The main advantage of rough set theory in data analysis is that it does not need any preliminary or additional information about data like probability distributions needed in statistics, basic probability assignments needed in evidence theory, a grade of membership or the value of possibility needed in fuzzy set theory.

Let us list a set of unique features which place the rough set approach at an advantageous position from the perspective of application. Being grounded in data it naturally can deal with (1) introduction of efficient algorithms for finding hidden patterns in data, (2) determination of optimal sets of data (data reduction), evaluation of the significance of data, (3) generation of sets of decision rules from data, (4) easy-to-understand formulation, (5) straightforward interpretation of obtained results, (6) suitability of many of its algorithms for parallel processing.

It is worthwhile to mention that rough sets play a crucial role in the development of granular computing (GrC) (Pedrycz et al. 2008). The extension of GrC to interactive granular computing (IGrC) requires generalization of the basic concepts such as complex granules (including both physical and abstract parts), information (decision) systems as well as methods of inducing hierarchical structures of information (decision) systems (Jankowski 2017; Jankowski et al. 2014b, 2015; Skowron and Jankowski 2016a, b, c; Skowron et al. 2012a, 2016) and interactions among information systems. The current research projects are aiming at developing foundations of IGrC based on the rough set approach in combination with other soft computing approaches, in particular with fuzzy sets. The approach is called interactive rough granular computing (IRGrC). In IRGrC computations are based on interactions in the physical world controlled by complex granules. IRGrC can be treated as the basis for developing: (1) Wisdom Technology, in particular for approximate reasoning (called adaptive judgment) about properties of interactive computations, (2) context inducing and discovery of structured objects, (3) reasoning about changes, (4) process mining (this research was inspired by Pawlak 1992), (5) perception based computing, (6) risk management in computational systems, etc.

Due to the space limitation we restrict to a few references of rough sets including two basic papers by Pawlak (1982, 1991), some survey papers (Pawlak and Skowron 2007; Skowron et al. 2015) and books (Chikalov et al. 2012; Pal et al. 2004; Skowron and Suraj 2013). The basic ideas of rough set theory and its extensions as well as many interesting applications can be found in a number of books, issues of the Transactions on Rough Sets, special issues of other journals, numerous proceedings of international conferences, and tutorials (see e.g., Chikalov et al. 2012; Kacprzyk and Pedrycz 2015; Pawlak and Skowron 2007; Skowron and Suraj 2013). The readers are referred to the cited books and papers, references in them as well as to the web pages www.roughsets.org, rsds.univ.rzeszow.pl. In this survey, we concentrate on a computational approach to rough sets rather than on the conceptual approach. Both of these approaches are present in works by Pawlak. These two approaches are characterized in Yao (2015).

In this paper we present discussion on some selected research directions of rough sets developed over the last 35 years, and we also outline some future perspectives of rough sets in relation to reaction systems (Ehrenfeucht et al. 2017; Ehrenfeucht and Rozenberg 2006) and IGrC. This paper summarizes and extends the aspects considered in Pawlak and Skowron (2007), Skowron et al. (2013, 2015) and Skowron and Suraj (2013).

## Rudiments of rough sets

The philosophy of rough set is founded on the assumption that with every object of the universe of discourse some information (data, knowledge) is associated. Objects characterized by the same information are indiscernible (similar) in view of the available information about them. The *indiscernibility relation* generated in this way is the mathematical basis of rough set theory. This understanding of indiscernibility is related to the idea of Gottfried Wilhelm Leibniz, according to him objects are indiscernible if and only if all available functionals take on them identical values (Leibniz’s Law of Indiscernibility: The Identity of Indiscernibles) (Leibniz 1686). However, in the rough set approach indiscernibility is defined relative to a given set of functionals (attributes).

Any set of all indiscernible (similar) objects is called an elementary set, and forms a basic granule (atom) of knowledge about the universe. An arbitrary union of some elementary sets is referred to as *crisp* (precise) set. If a set is not crisp then it is called *rough* (imprecise, vague). It is to be noted, that due to the computational complexity of searching for relevant crisp sets for the considered problem, the searching is usually restricted to a feasible subfamily of the family of all possible unions of elementary sets.

Consequently, each rough set has *borderline cases*, i.e., objects which cannot be classified with certainty as members of either the set or its complement. Obviously, crisp sets have no borderline elements at all.

Thus, the assumption that objects can be “seen” only through the information available about them leads to the view that knowledge has granular structure. Due to the granularity of knowledge, some objects of interest cannot be discerned, and appear as the same (or similar). As a consequence, vague concepts, in contrast to precise concepts, cannot be characterized in terms of only the elements belonging to the concept or satisfying the concept. Therefore, in the proposed approach, we assume that any vague concept is replaced by a pair of precise concepts—called the lower and the upper approximation of the vague concept. The lower approximation consists of all objects which definitely belong to the concept, and the upper approximation contains all objects which possibly belong to the concept. The difference between the upper and the lower approximation constitutes the boundary region of the vague concept. These approximations are two basic operations in rough set theory.

Hence, rough set theory expresses vagueness not by means of membership, but by employing a boundary region to a set. If the boundary region of a set is empty, it means that the set is crisp, otherwise the set is rough (inexact). A nonempty boundary region of a set indicates also the possibility that our knowledge about the set is not sufficient to define the set precisely.

In the literature one can find more details on different aspects of rough set approximations of vague concepts.

The starting point of rough set theory is the indiscernibility relation, which is generated from the information about objects of interest (defined later in this section as signatures of objects). The aim of indiscernibility relation is to express the fact that we are unable to discern some granules (or clusters) of objects, each having the same properties, based on the available information (or knowledge). This entails that, in general, we are unable to deal with each particular object separately; rather we can only consider granules (clusters) of indiscernible objects as a fundamental basis for the theory.

From a practical point of view, it is better to define basic concepts of this theory in terms of data. Therefore we will start our considerations from a data set called an *information system*.

Suppose we are given a pair ${\mathbb{IS}}= (U,AT)$ of non-empty, finite sets *U* and *AT*, where *U* is the *universe* of *objects*, and *AT* is a set consisting of *attributes*. Each attribute can be considered as a function $at{:}\,U \longrightarrow V_{at}$, where $V_{at}$ is the set of values for attribute *at*, called the *domain* of *at*. The pair ${\mathbb{IS}} = (U, AT)$ is called an *information system* (see e.g., Pawlak 1981). It is to be noted here that similar to the notion of information system, Barwise and Seligman (1997) have introduced a notion of classification.

Any information system can be represented by a data table with rows labeled by objects and columns labeled by attributes. Any pair (*x*, *at*), where $x\in U$ and $at\in AT$ defines the particular entry in the table indicated by the value *at*(*x*).

### **Definition 1**

Any subset $AT^{\prime }$ of *AT* determines a binary relation ${\mathcal {IND}}_{AT^{\prime }}\subseteq U\times U,$ called an *indiscernibility relation*, defined by

1 $$\begin{aligned} x\,{{\mathcal {IND}}_{AT^{\prime }}}\,y\,{\text{if}\,\text{and}\,\text{only}\,\text{if}}\,at(x) = at(y)\,{\text{for}\,\text{every}}\,at \in AT^{\prime }. \end{aligned}$$

Obviously, ${\mathcal {IND}}_{AT^{\prime }}$ is an equivalence relation. The set of all equivalence classes of ${\mathcal {IND}}_{AT^{\prime }}$, i.e., the partition determined by $AT^{\prime }$, will be denoted by $U/{{\mathcal {IND}}_{AT^{\prime }}}$, or simply $U/AT^{\prime };$ an equivalence class of ${\mathcal {IND}}_{AT^{\prime }}$, i.e., the block of the partition $U/AT^{\prime }$, containing *x* will be denoted as $[x]_{AT^{\prime }}$ (or more precisely $[x]_{{\mathcal {IND}}_{AT^{\prime }}}$) or $AT^{\prime }(x)$.

If $(x, y) \in {\mathcal {IND}}_{AT^{\prime }}$ we say that *x* and *y* are $AT^{\prime }$-*indiscernible*. Equivalence classes of the relation ${\mathcal {IND}}_{AT^{\prime }}$ (or blocks of the partition $U/AT^{\prime }$) are referred to as $AT^{\prime }$-*elementary sets* or $AT^{\prime }$-*elementary granules*. In the rough set approach, the elementary sets are the basic building blocks (concepts) of our knowledge about reality. The unions of $AT^{\prime }$-*elementary sets* are called $AT^{\prime }$-*definable sets*.

For $AT^{\prime }\subseteq AT$ we denote by $Inf_{AT^{\prime }}(x)$ the $AT^{\prime }$-*signature* of $x\in U$, which is represented by the set $\{(at, at(x)){:}\,at\in AT^{\prime }\}$. Let $Inf_{AT^{\prime }}(U)=\{Inf_{AT^{\prime }}(x){:}\,x \in U\}$. Then for any objects $x,y\in U$ the following equivalence holds: $x{\mathcal {IND}}_{AT^{\prime }}y$ if and only if $Inf_{AT^{\prime }}(x)= Inf_{AT^{\prime }}(y)$.

This indiscernibility relation is further used to define the basic concepts of rough set theory. Below we present some definitions for $AT^{\prime } \subseteq AT.$

### **Definition 2**

The following two operations on sets $X\subseteq U$, given by,

2 $$\begin{aligned} {\mathsf {LOW}}_{AT^{\prime }}(X)= \left\{ x \in U{:}\,[x]_{AT^{\prime }} \subseteq X \right\} , \end{aligned}$$

3 $$\begin{aligned} {\mathsf {UPP}}_{AT^{\prime }}(X)= \left\{ x \in U{:}\,[x]_{AT^{\prime }} \cap X \ne \emptyset \right\} , \end{aligned}$$

assign to every subset *X* of the universe *U*,  respectively two sets ${\mathsf {LOW}}_{AT^{\prime }}(X)$ and ${\mathsf {UPP}}_{AT^{\prime }}(X)$, called the $AT^{\prime }$-*lower* and the $AT^{\prime }$-*upper* *approximation* of *X*.

### **Definition 3**

The set

4 $$\begin{aligned} {\mathsf {BN}}_{AT^{\prime }}(X) = {\mathsf {UPP}}_{AT^{\prime }}(X) - {\mathsf {LOW}}_{AT^{\prime }}(X), \end{aligned}$$

is referred to as the $AT^{\prime }$-*boundary region* of *X*.

Any pair $\mathbb {AS}=(U,{\mathcal {IND}}_{AT^{\prime }}),$ where $AT^{\prime } \subseteq AT$, is called an *approximation space*. One can rewrite the above definitions relative to the approximation space $\mathbb {AS}.$

5 $$\begin{aligned} {\mathsf {LOW}}_{\mathbb {AS}}(X)&= \left\{ x \in U{:}\, [x]_{{\mathcal {IND}}_{AT^{\prime }}} \subseteq X \right\} , \nonumber \\ {\mathsf {UPP}}_{\mathbb {AS}}(X)&= \left\{ x \in U{:}\, [x]_{{\mathcal {IND}}_{AT^{\prime }}} \cap X \ne \emptyset \right\} ,\nonumber \\ {\mathsf {BN}}_{\mathbb {AS}}(X)&= {\mathsf {UPP}}_{{\mathcal {IND}}_{AT^{\prime }}}(X) - {\mathsf {LOW}}_{{\mathcal {IND}}_{AT^{\prime }}}(X). \end{aligned}$$

If the boundary region of *X* is the empty set, i.e., ${\mathsf {BN}}_{AT^{\prime }}(X) = \emptyset$, then the set *X* is *crisp* (*exact*) with respect to $AT^{\prime }$; in contrary, if ${\mathsf {BN}}_{AT^{\prime }}(X) \ne \emptyset$, the set *X* is referred to as *rough* (*inexact*) with respect to $AT^{\prime }$.

Therefore with every rough set we associate two *crisp* sets, called *lower* and *upper approximation*. Intuitively, the lower approximation of a set consists of all elements that *surely* belong to the set, and the upper approximation of the set constitutes of all elements that *possibly* belong to the set. The *boundary region* of the set consists of all elements that cannot be classified uniquely as belonging to the set or as belonging to its complement, with respect to the available knowledge. This is exactly the idea of vagueness proposed by Frege (1903).

Let us also observe that the definition of rough set starts with referring to data (knowledge), and hence it is *subjective*, in contrast to the definition of classical sets, which is in some sense an *objective* one.

Information systems with distinguished attributes (decisions) are called *decision systems*. More formally, a decision system is a tuple $\mathbb {DT} = (U, AT, d)$, where (*U*, *AT*) is an information system and $d\notin AT$ is a distinguished attribute $d{:}\,U \longrightarrow V_d$ called *decision attribute* or *decision function*. The set $V_d$ is the set of values for decision attribute *d*. Each value $v\in V_d$ defines a *decision class* $X_v=\{x\in U{:}\,d(x)=v\}$. Attributes from *AT* are called *conditional attributes* (or *conditions*).

## From partitions to coverings and beyond

Let us recall that a covering $\mathcal {C}$ of a nonempty (finite) set *U* is a family of nonempty subsets of *U* such that the union of this family is equal to *U*,  i.e., $\bigcup \mathcal {C}=U.$ A covering $\mathcal {C}$ of *U* is a partition if and only if for any $X,Y\in \mathcal {C}$ we have $X\cap Y= \emptyset$, if $X\ne Y.$

The original approach of Professor Pawlak was based on indiscernibility, defined by equivalence relations; more exactly on approximation spaces of the form (*U*, *IND*),  where *U* is a finite set and $IND\subseteq U\times U$ is an equivalence relation over *U*. Any such indiscernibility relation defines a partition of the universe of objects. Over the years many generalizations of this approach were introduced, many of which were based on coverings rather than partitions. In particular, one can consider similarity (tolerance) based rough set approach, binary relation based rough set approach, neighborhood and covering based rough set approach, dominance based rough set approach, hybridization of rough sets and fuzzy sets, and many others (see e.g., Kacprzyk and Pedrycz 2015; Skowron et al. 2015; Vluymans et al. 2015).

Let us consider an example of generalization of approximation space. A generalized approximation space^1^ can be defined by a tuple $\mathbb {AS}=(U,{\mathscr {I}},\nu )$ where ${\mathscr {I}}$ is the *uncertainty function* defined on *U* with values in the powerset ${\mathscr {P}}(U)$ of *U*, ${\mathscr {I}}({x})$ is considered to be the *neighboorhood* of *x*. W assume $x\in {\mathscr {I}}(x)$ for any $x\in U.$ The *inclusion function* $\nu$ is defined on the Cartesian product ${\mathscr {P}}(U)\times {\mathscr {P}}(U)$, takes values in the interval [0, 1] measuring the degree of inclusion of two sets (Skowron and Stepaniuk 1996). It should be noted that the uncertainty function ${\mathscr {I}}$ defines, in general, a covering $\{{\mathscr {I}}(x){:}\,x\in U\}$ of *U* rather than a partition of *U*.

The lower and upper approximation operations can be defined in $\mathbb {AS}$ by

6 $$\begin{aligned} {\mathsf {LOW}}_{\mathbb {AS}}(X)= & {} \left\{ x \in U{:}\, \nu ({\mathscr {I}}({x}),X)=1 \right\} , \end{aligned}$$

7 $$\begin{aligned} {\mathsf {UPP}}_{\mathbb {AS}}(X)= & {} \left\{ x \in U{:}\,\nu ({\mathscr {I}}({x}),X) > 0\right\} . \end{aligned}$$

In the approach formulated by Pawlak, ${\mathscr {I}}({x})$ is equal to the equivalence class $[x]_{AT^{\prime }}$ of the indiscernibility relation ${\mathcal {IND}}_{AT^{\prime }}$; in case of tolerance (similarity) relation $\tau \subseteq U\times U$ (Polkowski et al. 1995) we take ${\mathscr {I}}({x})=[x]_{\tau } = \{y\in U{:}\,x\ \tau \ y\}$, i.e., ${\mathscr {I}}({x})$ is equal to the tolerance class of $\tau$ with respect to *x*. The standard way od defining rough inclusion relation, denoted by $\nu _{SRI},$ for $X,Y\subseteq U$ is as follows.^2^

8 $$\begin{aligned} \nu _{SRI}(X,Y)= {\left\{ \begin{array}{ll} \frac{\left| {X\cap Y}\right| }{\left| {X}\right| }, &{}\quad {\mathrm{if}}\,X\,{\mathrm{is}}\,{\text{non-empty}},\\ 1, &{}\quad {\mathrm{otherwise}}. \end{array}\right. } \end{aligned}$$

For applications it is important to come up with some constructive definitions of ${\mathscr {I}}$ and $\nu$.

One should note that dealing with coverings requires solving several new algorithmic problems such as selection of family of definable sets or resolving problems with selection of relevant definition of approximation of sets among many possible ones. One should also note that for a given problem (e.g., classification problem) one should discover the relevant covering for the target classification. In the literature there are numerous papers dedicated to theoretical aspects of the covering based rough set approach. However, still much more work should be done on rather hard algorithmic issues for the discovery of relevant covering.

Another issue, to be solved, is related to inclusion measures. Parameters of such measures are tuned to induce the high quality of approximations. Usually, this is done on the basis of the minimum description length principle (MDL). In particular, approximation spaces with rough inclusion measures have been investigated. This approach was further extended to rough mereological approach. More general cases of approximation spaces with rough inclusion were also discussed in the literature including approximation spaces in GrC. Finally, the approach for ontology approximation used in hierarchical learning of complex vague concepts (Skowron and Suraj 2013) is especially worthwhile to mention.

One of the present challenges is to extend the rough set approach of approximations, based on GrC, in the context of IGrC, which incorporates interactions with the environment. In particular, in intelligent systems (IS) and complex adaptive systems (CAS) in order to control computation and agent needs to invent adaptive strategies for approximation of decision functions. So, this direction of research is of great importance in applications.

It is worthwhile mentioning that over the years the definition of information system is changing. In particular, in Skowron and Dutta (2017) any attribute *at* is linked not only with the value set $V_{at}$ but also with a relational structure ${\mathcal R}_{at}$ over $V_{at}$. This addresses cases such as discretization or preferences over value sets of attributes (see e.g., Greco et al. 2004; Nguyen 2006). In this case one should also consider that a set of formulas ${\mathcal F}_{at}$, linked to the attribute *at* is interpreted over ${\mathcal R}_{at}$. From this set ${\mathcal F}_{at}$, some formulas may be used as constraints in the contexts of feature extraction problem. Recently, it was also emphasized (see e.g., Jankowski 2017; Jankowski et al. 2014b, 2015; Skowron and Jankowski 2016a, b, c; Skowron et al. 2012a, 2016) that for many applications, information systems should be considered as open objects (complex granules) rather than closed objects (complex granules), where they are open to interact with the environment, consisting physical objects as well as other information systems grounded on physical objects too. One of the consequences of this point of view is the necessity of developing methods for controlling these interactions toward achieving the needs of agents.

## Rough sets and induction

Rough set based approach has strong potential to model inductive reasoning. Inducing classifiers or clusters using rough set based methods is one such example. In this section, we present an illustrative example of the rough set approach towards induction of concept approximations. The approach can be generalized considering inductive extensions of approximation spaces.

Let us consider the problem of approximation of concepts over a universe $U^{\infty }.$ We assume that the concepts are perceived only through some subsets of $U^{\infty }$, called samples. This is a typical situation in the machine learning, pattern recognition, or data mining approaches (Cios et al. 2007).

We assume that an information system ${\mathbb{IS}}=(U,AT)$ is given (where $U\subseteq U^{\infty }$), and that for some $C\subseteq U^{\infty }$ there is a set $\varPi _U(C)=C\cap U$. In this way, we obtain a decision system $\mathbb {DT}_d=(U,AT,d)$, where $d(x)= 1$ if $x\in \varPi _U(C)$ and $d(x)= 0$, otherwise.

We would like to illustrate how from the decision function *d* defined over *U*,  one can induce a decision function $\mu _C,$ defined over $U^{\infty }(\supset U),$ with values in the interval [0, 1]. This can be treated as an approximation of the characteristic function of *C*.

Let us assume that $RULES(\mathbb {DT}_d)$ is a set of decision rules induced by some rule generation method from $\mathbb {DT}_d$. For any object $x \in U^{\infty }$, let

$$\begin{aligned} MatchRules(\mathbb {DT}_d, x) \end{aligned}$$

be the set of rules that is supported by *x*.

Now, the rough membership function $\mu _{C}: U^{\infty } \rightarrow [0,1]$, approximating the characteristic function of *C*, can be defined as follows.

1. Let ${R}_{k}(x)$ (where $k=0,1$), for $x\in U^{\infty }$ be the set of all decision rules from $MatchRules(\mathbb {DT}_d, x)$ with right hand side $d=k$, where $d=1$ denotes that the rule *r* is voting for *C* and $d=0$ denotes that the rule *r* is voting against *C*, respectively.
2. We define real values $w_{k}(x)$, where $w_{1}(x)$ is called the weight “for” and $w_{0}(x)$ the weight “against” membership of the object *x* in *C*, respectively, and $w_{k}(x) = \sum _{r\in R_k(x)} strength(r);$ here *strength* is a normalized function depending on *length*, *support*, *confidence* of decision rules and on some global information about the decision system $\mathbb {DT}_d$ such as the size of the decision system or the class distribution.
3. Finally, one can define the value of $\mu _{C}(x)$ in the following way: $\mu _{C}(x)$ is undefined if $\max (w_{1}(x),w_{0}(x)) < \omega$; $\mu _{C}(x)=0$ if $w_{0}(x) - w_{1}(x) \ge \theta$ and $w_{0}(x)> \omega$; $\mu _{C}(x)=1$ if $w_{1}(x) - w_{0}(x) \ge \theta$ and $w_{1}(x)> \omega$ and $\mu _{C}(x)=\frac{\theta + (w_{1}(x) - w_{0}(x))}{2\theta }$, otherwise, where $\omega , \theta$ ($\omega \ll \theta$) are the parameters set by the user.

**Fig. 1:**
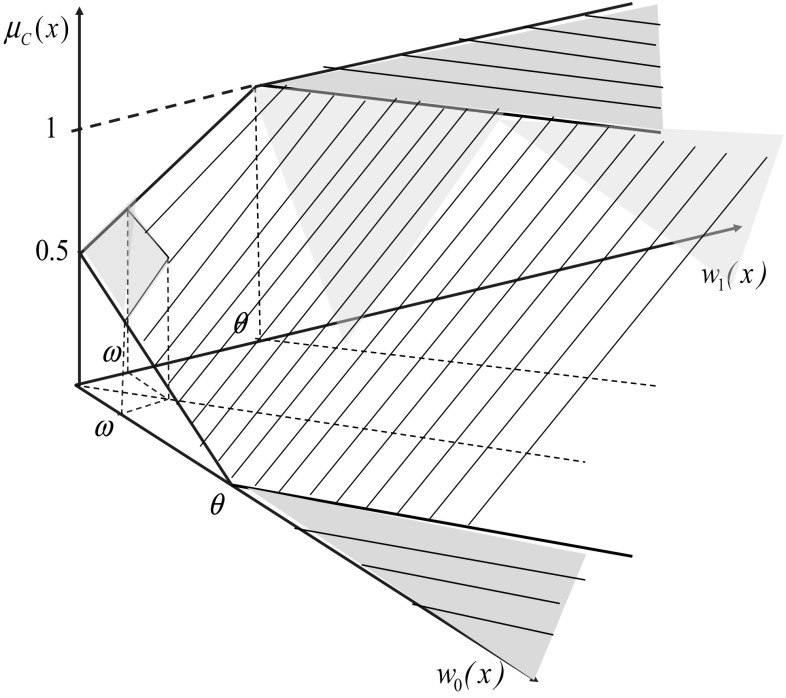
Exemplary rough membership function $\mu _C$

In Fig. 1 the induced exemplary rough membership function $\mu _C$ is presented.

For computing the value $\mu _C(x)$ for $x\in U^{\infty }$ the user should select a strategy resolving the conflict between the votes “for” and “against” the membership of *x* in *C*. The degree of these conflicts are represented by values $\mu _{1}(x)$ and $\mu _{0}(x)$, respectively. Note that for some *x* due to the small differences between these values the selected strategy may not produce the definite answer, and these cases will create the boundary region.

We can now define the lower approximation, the upper approximation and the boundary region of the concept *C*, relative to the induced rough membership function, $\mu _C$ as follows

9 $$\begin{aligned}&LOW(C,\mu _C) = \left\{ x\in U^{\infty }{:}\,\mu _C(x)=1\right\} , \nonumber \\&UPP(C,\mu _C) = \left\{ x\in U^{\infty }{:}\,\mu _C(x)>0 \,{\text{or}}\,\mu _C(x)\,{\text{is}\,\text{undefined}}\right\} , \nonumber \\&BN(C,\mu _C) = UPP(C,\mu _C) {\setminus } LOW(C,\mu _C). \end{aligned}$$

The whole procedure can be generalized for the case of approximation of more complex information granules than simple concepts.

## Boolean reasoning and scalability

Solutions for many algorithmic problems related to rough sets were proposed using the (approximate) Boolean reasoning approach (Blake 1937; Boole 1948, 1954; Brown 1990; Skowron and Suraj 2013). Some progress was also made in developing methods scalable for large data sets. In this section we present discussion on some applications of Boolean reasoning approach for solving different problems using rough sets.

The discernibility relations are closely related to indiscernibility and belong to the most important relations considered in rough set theory. However, it is to be noted that the discernibility relation is not always defined as the complement of the indiscernibility relation. Tools for discovering and classifying patterns are based on *reasoning schemes* rooted in various paradigms. Such patterns can be extracted from data by means of methods using Boolean reasoning and the notion of discernibility.

The ability to discern between perceived objects is important for constructing reducts, decision rules or decision algorithms. In the standard approach, the discernibility relation ${\mathcal {DIS}}_{AT^{\prime }}\subseteq U\times U$ is defined by $x {\mathcal {DIS}}_{AT^{\prime }} y$ if and only if it is not that $x {\mathcal {IND}}_{AT^{\prime }} y,$ i.e., $AT^{\prime }(x)\cap AT^{\prime }(y)= \varnothing$, where $AT^{\prime }(x)$, $AT^{\prime }(y)$ are neighborhoods of *x* and *y*, respectively. However, this is not the case for generalized approximation spaces.

The idea of Boolean reasoning is based on construction of a corresponding Boolean function $f_{P}$ for a given problem *P* having the following property: the solutions for the problem *P* can be decoded from prime implicants of the Boolean function $f_{P}$ (Brown 1990; Nguyen 2006; Skowron 2000). Let us mention that to solve real-life problems it is necessary to deal with Boolean functions of large size.

A successful methodology based on the discernibility of objects and Boolean reasoning has been developed for computing many important factors of applications. These applications include generation of reducts and their approximations, decision rules, association rules, discretization of real-valued attributes, symbolic value grouping, searching for new features defined by oblique hyperplanes or higher order surfaces, pattern extraction from data as well as conflict resolution or negotiation (see e.g., Pawlak and Skowron 2007; Skowron and Suraj 2013).

Most of the problems related to generation of the above mentioned aspects are NP-complete or NP-hard. However, it was possible to develop efficient heuristics providing suboptimal solutions of the problems. The results of experiments on many data sets are very promising. They show very good quality of solutions generated by the heuristics in comparison with other methods reported in literature (e.g., with respect to the classification quality of unseen objects). Moreover, they are very efficient from the point of view of time that is necessary for computing the solution. Many of these methods are based on discernibility matrices. However, it is possible to compute the necessary information about these matrices without their explicit construction (i.e., by sorting or hashing original data).

The considered methodology makes it possible to construct heuristics having a very important *approximation property* which can be formulated as follows: expressions, called *approximate implicants*, generated by heuristics, that are *close* to prime implicants, define approximate solutions for the problem (Nguyen 2006).

Mining large data sets is one of the biggest challenges in Knowledge Discovery and Databases (KDD). In many practical applications, there is a need of data mining algorithms running on terminals of possibly distributed database systems where the only access to data is enabled by SQL queries or NoSQL operations.

Let us consider two illustrative examples of problems for large data sets: (1) searching for short reducts, and (2) searching for best partitions defined by cuts on continuous attributes. In both cases, the traditional implementations of rough sets and Boolean reasoning based methods characterizes the high computational cost. The critical factor for time complexity of algorithms solving the discussed problems is the number of data access operations. Fortunately some efficient modifications of the original algorithms were proposed by relying on concurrent retrieval of higher level statistics which are sufficient for the heuristic search of reducts and partitions (see e.g., Pawlak and Skowron 2007; Skowron and Suraj 2013). The rough set approach was also applied in development of other scalable big data processing techniques (e.g., Slezak and Eastwood 2009).

## Rough sets: some future challenges from the point of view of applications

Complex adaptive systems (CAS) are made up of multiple interacting elements, and have the capacity to change themselves and learn from experience. The key problems of complex systems are difficulties with their formal modeling and simulation.^3^ Some approaches to modeling CAS are based on agent-based models and/or complex network-based models (see e.g., Meia et al. 2015; Yang and Shan 2008).

Decision support in the context of CAS (Holland 2014; Valiant 2013; Yang and Shan 2008) requires identification of the relevant computation models as well as methods for incorporating reasoning behind computations performed by agents. Agents perform computations on complex objects (e.g., behavioral patterns, classifiers, clusters, structural objects, sets of rules, aggregation operations, approximate reasoning schemes). In granular computing (GrC), all such constructed and/or induced objects are called granules. To model interactive computations (Goldin et al. 2006) performed by agents, the existing GrC approach to interactive granular computing (IGrC) was extended by introducing interactions among different parts of *complex granules* (*c-granules* or *granules*, for short).

The IGrC approach is an extension of the joint research with Andrzej Jankowski (Jankowski and Skowron 2007, 2008, 2009; Jankowski et al. 2014a, 2015; Nguyen et al. 2010; Skowron and Jankowski 2016a; Skowron et al. 2012b, 2013, 2016). This is a step towards realization of the Wisdom Technology (WisTech) program (Jankowski 2017; Jankowski and Skowron 2007, 2008, 2009; Skowron et al. 2012a) in combination with IGrC, and is developed over years of experiences, based on the work on different real-life projects. The developed model is called the Wistech IGrC model.

Other issues such as evolution of communication language of agents and risk management in interactive computations will be discussed in more detail in our next papers (see also Jankowski 2017).

We would like to emphasize that still much more work should be done to develop approximate reasoning methods about complex vague concepts for making progress in development of IS or CAS. This idea was very well expressed by Professor Leslie Valiant^4^:

A fundamental question for artificial intelligence is to characterize the computational building blocks that are necessary for cognition.

In IGrC, the computational building blocks are represented by complex granules (c-granules, for short) which are used to model computations in IS or CAS.

In the following section we present some intuitive explanations concerning c-granules and IGrC. For more details the readers are referred to Jankowski (2017), Jankowski et al. (2014b, 2015), Skowron and Jankowski (2016a, b, c) and Skowron et al. (2012a, 2016). We begin with a discussion on modeling complex states and transition relations on such states.

### Modeling of complex states and transition relations on complex states

In this section, we discuss two fundamentally different styles of modeling. In the first case, the models are designed by humans in the world of mathematics and next they are verified in the physical reality. In the second case, models are learned through interactions with the environment and they are continuously tuned using new acquired data and accumulated knowledge. After satisfactory interactions with the environment the model, which was not available to the designer a priori, rather is learned with time, provides a representation of the agent’s environment. In this regard, the modeling needs to be based on the information acquired by agent-environment interaction. We discuss the first step in this direction by linking reaction systems with rough sets.

We start by quoting Brooks (1975).

Mathematics and the physical sciences made great strides for three centuries by constructing simplified models of complex phenomena, deriving, properties from the models, and verifying those properties experimentally. This worked because the complexities ignored in the models were not the essential properties of the phenomena. It does not work when the complexities are the essence.

Taking into account the above opinion one may expect that the models of transition relations on states of complex systems designed by humans may not reflect the dynamics of complex systems. Usually we have only a partial, imprecise or imperfect information about states. Moreover, questions related to perception arise too. In particular, these can be questions about the perception of states perceived by agents performing computations. The answers to such questions depend on the understanding of interactions of agents with the complex system embedded in the environment. Through interactions the agents can try to get satisfactory information for performing relevant actions toward achieving their goals. Here, one should resolve the problems related to understanding interactions of physical objects to gain proper information about the environment in which the tasks are performed. Some progress in this direction has been made in the context of IGrC (see e.g., Jankowski 2017; Jankowski et al. 2014b, 2015; Skowron and Jankowski 2016a, b, c; Skowron et al. 2012a, 2016 and the following sections).

In this section, we restrict our discussion to two approaches for modeling processes. The first one was developed for reaction systems, proposed for modeling chemical and/or biological processes (Ehrenfeucht and Rozenberg 2006, 2007, 2009). We call them exact models. The second one is based on the rough set approach (Pawlak 1982, 1991; Pawlak and Skowron 2007). In the case of the rough set approach the transition relation is learned from data gathered from the result of interactions of the ‘agent’ with the phenomena in the environment. The induced models are evolving with time using adaptive strategies.

In the following subsections we discuss an application of realistic modeling using the framework of rough sets (Pawlak 1982, 1991; Pawlak and Skowron 2007; Skowron and Nguyen 2013) to reaction systems which originated as a pure mathematical model of interactions of biochemical reactions in the living cell (Brijder et al. 2011; Ehrenfeucht et al. 2012, 2017; Ehrenfeucht and Rozenberg 2006; Salomaa 2012). For more details the reader is referred to Dutta et al. (2018).

#### Reaction systems

In this subsection we recall some basic notions concerning reaction systems (mostly taken from Ehrenfeucht and Rozenberg 2006; Ehrenfeucht et al. 2017). The original motivation behind reaction systems was to model interactions between biochemical reactions in the living cell. Therefore, the formal notion of reaction reflects the basic intuition behind biochemical reactions. A biochemical reaction can take place if all of its reactants are present in a given state and none of its inhibitors is present. When a reaction takes place, it creates its products. This leads to the following definitions.

##### **Definition 4**

A *reaction* is a triplet $a = (R_a,I_a,P_a)$, where $R_a, I_a, P_a$ are finite nonempty sets with $R_a \cap I_a = \emptyset$. If *S* is a set such that $R_a, I_a, P_a\subseteq S$, then *a* is a reaction in *S*.

The sets $R_a, I_a, P_a$, are called the reactant set of *a*, the inhibitor set of *a*, and the product set of *a*, respectively. Clearly, since $R_a, I_a$ are disjoint and nonempty, if *a* is a reaction over *S*, then $|S|\ge 2.$ We will use *rac*(*S*) to denote the set of all reactions over *S*.

The enabling of a (biochemical) reaction in the given state of a biochemical system and the resulting state transformation are defined as follows.

##### **Definition 5**

Let *T* be a finite set

- Let *a* be a reaction. Then *a* is *enabled by* *T*, denoted by $en_a(T )$, if $R_a\subseteq T$ and $I_a\cap T = \emptyset$. The result of *a* on *T*, denoted by $res_a(T)$, is defined by: $res_a(T) = P_a$ if $en_a(T)$ and $res_a(T) = \emptyset$, otherwise.
- Let *A* be a finite set of reactions. The *result of* *A* on *T*,  denoted by $res_A(T)$, is defined by: $res_A(T) = \bigcup _{a\in A} res_a(T)$.

The intuition behind a finite set *T* is that of a state of a biochemical system, i.e., a set of biochemical entities present in the current biochemical environment. Thus a single reaction *a* is enabled by state *T* if *T* separates $R_a$ from $I_a$, i.e., $R_a\subseteq T$ and $I_a\cap T = \emptyset .$ When *a* is enabled by *T*, then its result on *T* is just $P_a.$ For a set *A* of reactions, its result on *T* is cumulative, i.e., it is the union of the results of all individual reactions from *A*. Since reactions which are not enabled by *T* do not contribute to the result of *A* on *T*,  $res_A(T)$ can be defined by

$$\begin{aligned} res_A(T)=\bigcup \left\{ res_a(T ) | a\in A\,{\text{ and }}\,en_a(T)\right\} . \end{aligned}$$

Now the central notion of a reaction system is defined as follows.

##### **Definition 6**

A *reaction system* is an ordered pair ${\mathcal {A}} = (S,A),$ where *S* is a finite set such that $|S|\ge 2$ and $A\subseteq rac(S)$ is a nonempty set of reactions in *S*.

Thus a reaction system is basically a finite set of reactions over a set *S*, which is called the *background set of* ${\mathcal {A}}$ and its elements are called *entities*. The *result function* of ${\mathcal {A}}$, $res_{{\mathcal {A}}}{:}\,2^S \longrightarrow 2^S$ is defined by $res_{{\mathcal {A}}}= res_A.$

The behaviour of a reaction system (which results from the interactions between its reactions) is determined by its dynamic processes which are formally defined as follows.

##### **Definition 7**

Let ${\mathcal {A}} = (S,A)$ be a reaction system and let $n \ge 1$ be an integer. An (*n*-step) *interactive process in* *A* is a pair $\pi = (\gamma , \delta )$ of finite sequences such that $\gamma = C_0, \ldots ,C_n$ and $\delta = D_0, \ldots , D_n$, where $C_0, \ldots , C_n, D_0,\ldots , D_n \subseteq S$, and $D_i = res_{{\mathcal {A}}}(D_{i-1} \cup C_{i-1})$ for all $i \in \{1, \ldots , n\}.$

The sequence $\gamma$ is the *context sequence of* $\pi$ and the sequence $\delta$ is the *result sequence of* $\pi .$. Then, the sequence $\tau = W_0, W_1, \ldots , W_n$ defined by $W_i = C_i \cup D_i$ for all $i \in \{0, \ldots , n\}$ is the *state sequence of* $\pi$ with $W_0 = C_0$ called the *initial state of* $\pi$ (and of $\tau$). If $C_i \subseteq D_i$ for all $i \in \{1, \ldots , n\}$, then we say that $\pi$ (and $\tau$) is context-independent. Note that we can assume then that $C_i = \emptyset$ for all $i \in \{1, \ldots , n\}$ without changing the state sequence.

Thus, an interactive process begins in the initial state $W_0=C_0\cup D_0$. The reactions from *A* enabled by $W_0$ produce the result $D_1$ which together with $C_1$ forms the successor state $W_1=C_1\cup D_1.$ The iteration of this procedure determines $\pi$: for each $i\in \{0,\ldots , n-1\}$, the successor of state $W_i$ is determined by $W_{i+1}=C_{i+1}\cup D_{i+1},$ where $D_{i+1}=res_{{\mathcal {A}}}(W_i).$

The context sequence formalizes the intuition that, in general, a reaction system is not a closed system and so its behavior is influenced by its “environment.” Note that a context-independent state sequence is determined by its initial state $W_0$ and the number of steps (*n*). In general, for an *n*-step interactive process $\pi$ of ${\mathcal {A}},$ $\pi$ is determined by its context sequence and *n*.

Also, in a context-independent state sequence $\tau = W_0, \ldots ,W_i,W_{i+1}, \ldots , W_n$, during the transition from $W_i$ to $W_{i+1}$ all entities from $W_i - res_{{\mathcal {A}}}(W_i)$ vanish. This reflects the assumption of *no permanency*: an entity from a current state vanishes unless it is produced/sustained by *A*. Clearly, if $\pi$ is not context-independent, then an entity from a current state $W_i$ can be also sustained (thrown in) by the context ($C_{i+1}$). This feature is also a major difference with standard models of concurrent systems such as Petri nets (see e.g., Reisig 2013).

#### Rough set-based modeling of complex states and transition relations

In the framework of reaction systems one uses statements like “*a system is in a state*” without being concerned with the questions such as *how this “being in a state” is perceived?* However, such questions are relevant from the practical/applied points of view, because due to the complex nature of physical systems only a partial, incomplete information about their states may be perceived. The rough set approach seems more realistic, in this regard, as it address the problem by assuming that states are perceived through attributes.

In the previous subsection we have recalled a mathematical model of reaction system. Now, we will discuss how to modify this model, so as to make it closer to the physical reality.

**Fig. 2:**
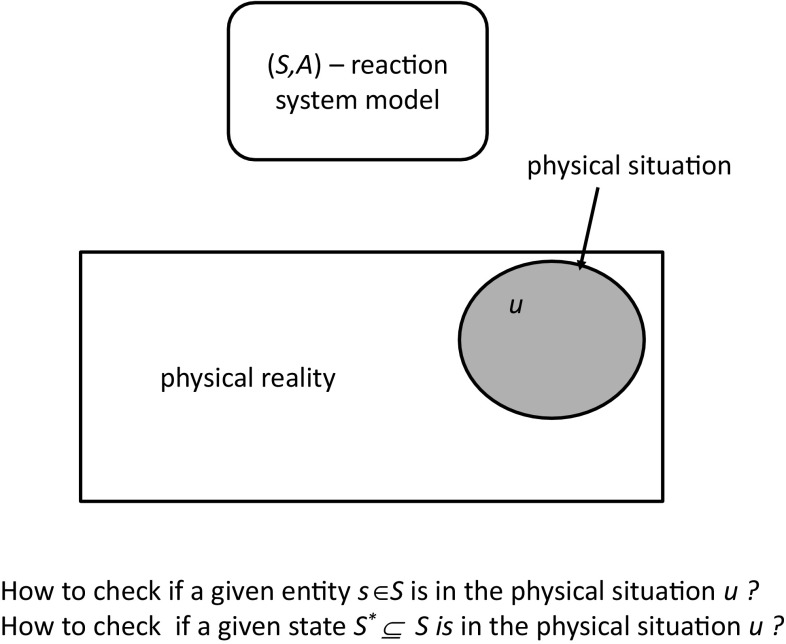
Reaction system and physical reality

**Fig. 3:**
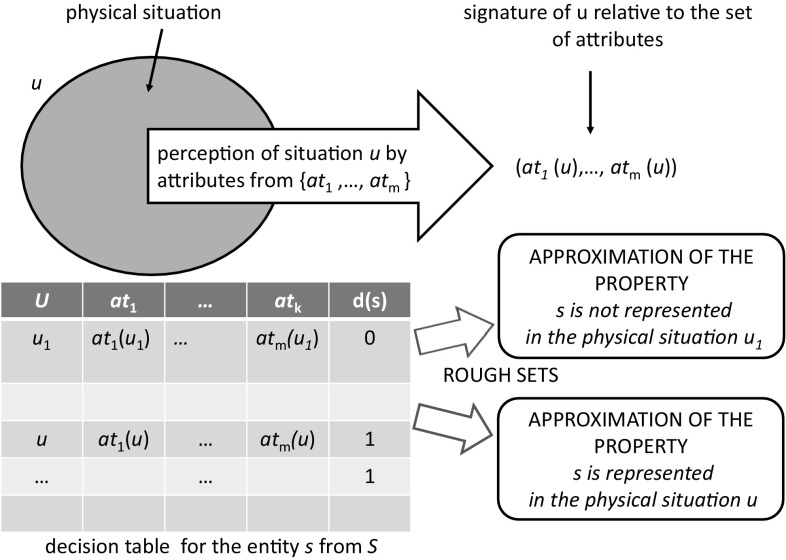
Approximation of basic concepts related to the relationship of reaction systems and physical reality

Let us assume that in the physical reality we can identify physical situations (see Fig. 2) and perceive them by some attributes (see Fig. 3). One can collect the results of perceived situations in the form of a data table to which a new column is added reflecting the domain expert’s opinion concerning the question whether or not a given entity *s* is represented in the perceived physical situation *u* (see Fig. 3). We will discuss now how rough sets can be used to approximate properties such as “a given entity is represented in a perceived situation” or “a given state of reaction system is represented in a perceived situation.” Then, we illustrate how one could check if the proposed model of reaction system is consistent with the perceived data.

Let ${\mathcal {A}}=(S,A)$ be a reaction system. For each entity $s\in S$, we consider a decision system $\mathbb {DT}(s) = (U, AT(s), d(s))$, where *U* is a sample from the space of physical objects perceived by attributes from *AT*(*s*). Accordingly, the elements of *U* are called *physical situations* (or *situations*, for short). The binary decision *d*(*s*) is the characteristic function of the concept “*entity s is represented in the situation u*”, where $u\in U.$ This induces the decision class $C(s)=\{u\in U| d(s)(u)=1 \}$ corresponding to this concept. *C*(*s*) consists of all situations from *U* which include a physical object representing the given entity *s*. The decision class *C*(*s*) can be approximated using the information system ${\mathbb{IS}}(s)= (U, AT(s))$, i.e., by the rough set methodology one can consider now the lower approximation ${\mathsf {LOW}}_{AT(s)}(C(s))$, the upper approximation ${\mathsf {UPP}}_{AT(s)}(C(s))$, and the boundary region ${\mathsf {BN}}_{AT(s)}(C(s)).$ These three components of approximation describe the result of perception of the property “*entity s is represented in situation u*.” In order to represent the results of approximating *C*(*s*) by these three components, one can define the generalized decision $\delta _{C(s)}$ for the decision class *C*(*s*) as follows: $\delta _{C(s)}(u)=1$ for $u\in {\mathsf {LOW}}_{AT(s)}(C(s))$, $\delta _{C(s)}(u)=0$ for $u\in U{\setminus } {\mathsf {UPP}}_{AT(s)}(C(s))$, and $\delta _{C(s)}(u)=\{0,1\}$ for $u\in {\mathsf {BN}}_{AT(s)}(C(s))$. This function can be extended to the indiscernibility classes of states or to the signatures of states by setting $\delta _{C(s)}([u]_{AT(s)})= \delta _{C(s)}(u)$ and $\delta _{C(s)}(Inf_{AT(s)}(u))= \delta _{C(s)}(u)$. For the sake of not complicating the notation, these extensions are also denoted by $\delta _{C(s)}$.

In order to express how a set of entities $\hat{S} \subseteq S$, i.e., a state of the system ${\mathcal {A}}$, is perceived we need to consider an aggregation of decision systems $\mathbb {DT}(s)$, for $s\in \hat{S}$. The result of this aggregation can also be defined as a decision system in the following way.

Let $\hat{S}=\{s_1, \ldots , s_k \}$ and $\mathbb {DT}(s_i) = (U, AT(s_i), d(s_i)),$ for $i=1,\ldots , k.$ We set then:

- $U(\hat{S})=\{\hat{v}(u){:}\,u\in U \}$, where for each $u\in U$, $\hat{v}(u)=(v_1(u), \ldots , v_k(u))$, where $v_i(u)=Inf_{AT(s_i)}(u)$, for $i=1,\ldots , k$ (i.e., $v_i(u)$ is the signature of *u* relative to the attributes from $AT(s_i)$),
- $AT(\hat{S})$ consists of attributes which are the characteristic functions of the lower approximations, the upper approximations, and the boundary regions of the decision classes $C(s_i)$ relative to the set of attributes $AT(s_i)$ from $\mathbb {DT}(s_i)$ for $i=1,\ldots , k$ extended to $U(\hat{S})$. More formally,
  $$\begin{aligned} AT(\hat{S})= \bigcup _{i=1,\ldots , k} \left\{ \chi _{{\mathsf {LOW}}_{AT(s_i)}(C(s_i))}, \chi _{{\mathsf {UPP}}_{AT(s_i)}(C(s_i))}, \chi _{{\mathsf {BN}}_{AT(s_i)}(C(s_i))}\right\} , \end{aligned}$$
  where
  1. $\chi _{{\mathsf {LOW}}_{AT(s_i)}(C(s))}(\hat{u})= 1$ if and only if $v_i(u)\in {\mathsf {LOW}}_{AT(s_i)}(C(s_i))$,
  2. $\chi _{{\mathsf {UPP}}_{AT(s_i)}(C(s_i))}(\hat{u})= 1$ if and only if $v_i(u)\in {\mathsf {UPP}}_{AT(s_i)}(C(s_i))$,
  3. $\chi _{{\mathsf {BN}}_{AT(s_i)}(C(s_i))} (\hat{u}) =\{0,1\}$ if and only if $v_i(u)\in {\mathsf {BN}}_{AT(s_i)}(C(s_i))$, and
- $d(\hat{S})$ to be the (generalized) decision defined by: for $u\in U,$
  $$\begin{aligned}d(\hat{S})(\hat{v}(u))= (\delta _{C(s_1)}(v_1(u)), \ldots , \delta _{C(s_k)}(v_k(u))). \end{aligned}$$
  The value $d(\hat{S})(\hat{v}(u))$ of the generalized decision $d(\hat{S})$ represents the result of perception of the property “*entities from* $\hat{S}$ *are represented in the current situation u*.” Note that for some entities the perception may not give a decision with certainty about their representation in the current state due to uncertainty in perceiving the physical situations through the available attributes.

Finally, we define the decision system $\mathbb {DT}(\hat{S})$ (which is an aggregation of decision systems $\mathbb {DT}(s_i),$ for $i=1,\ldots , k$) by

$$\begin{aligned} \mathbb {DT}(\hat{S})=(U(\hat{S}),AT(\hat{S}),d(\hat{S})). \end{aligned}$$

It is needed to point out here that other sorts of aggregations can also be defined using operations of join with constraints (Skowron and Stepaniuk 2005). They can be used to extract subsets of objects from $\mathbb {DT}(\hat{S})$ satisfying some constraints for which the tested hypothetical laws are true.

By selecting relevant sets of entities (i.e., relevant states) of a given reaction system one may consider modeling properties such as “*the inhibitors from the set* $I_a$ *are not represented in the current situation u*” as well as the property that this condition is true for all considered reactions. One may then aggregate decision (information) systems corresponding to these properties in order to represent the combined result of perception for all these properties. Analogously, it is possible to obtain decision systems representing properties such as “*product p from* $P_a$ *is represented in the current situation u*”, “*all products from* $P_a$ *are represented in the current situation u*”, and “*all products from* $P_a$ *are represented in the current situation u for all considered reactions a*.”

One can also consider aggregation of the already constructed decision systems into a decision system over pairs of objects with the first component of a pair representing the current physical situation and the second component describing the results of the reactions taking place in this situation. Through such systems one can construct a set of rules (local logic, a view of knowledge represented in the system) describing properties of the second component induced from the properties of the first component. For example, the following rule (written in an informal way) can be considered as a ‘justifying criterion’ (to some satisfactory degree) for the discussed exact model for reaction systems: 
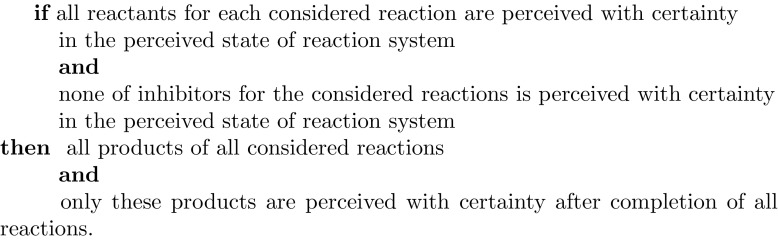


In this rule the term “*with certainty*” means that the considered situation belongs to the lower approximation of the relevant region. Thus, one may write this rule formally using formulas expressing the relevant approximated regions. Then, the validity of this rule may be checked in a given data table (information system).

Such rules can be rewritten in a more formal way using definable regions of approximation (i.e., unions of the indiscernibility classes) as follows.

Let for a reaction system ${\mathcal {A}}=(S,A):$

- *x* be the information perceived about the physical situation before performing reactions from ${\mathcal {A}},$ i.e., the (disjoint) union of signatures over the attribute sets of information systems corresponding to reactants, inhibitors, and products, respectively,
- *y* be the information perceived about the physical situation obtained after performing reactions from ${\mathcal {A}}$ in the physical situation represented by *x*,
- $R_a=\{r_1, \ldots , r_{k_a} \}$ be the reactant set of $a\in A$,
- $I_a=\{i_1, \ldots , i_{s_a} \}$ be the inhibitor set of $a\in A$,
- $P_a=\{p_1, \ldots , p_{n_a} \}$ be the product set of $a\in A$, and
- $$P= \bigcup _{a\in A} P_a$$.

Then one can formally express the rules and meta-rules for different levels of a reaction system as follows:

$$\begin{aligned}&en_a(x) \longrightarrow \bigwedge _{1\le l \le n_a} \chi _{ {\mathsf {LOW}}_{AT(p_l)}(C(p_l))}(y)=1,\,{\text{for}\,\text{each}}\,a \in A,\\&\chi _{{\mathsf {LOW}}_{AT(p)}(C(p))}(y)=1 \longrightarrow \bigvee _{a \in A} \left( en_a(x)\ \& \ p\in P_a\right) ,\,{\text{for}\,\text{each}}\,p \in P, \end{aligned}$$

where, for each $a\in A,$

$$\begin{aligned} en_a(x) \equiv \left[ \bigwedge _{1\le i \le k_a} \chi _{ {\mathsf {LOW}}_{AT(r_i)}(C(r_i))}(x)=1\ \& \bigwedge _{1\le j \le s_a} \chi _{{\mathsf {LOW}}_{AT(i_j)}(C(i_j))}(x)=0 \right] . \end{aligned}$$

The validity of the above rules in the corresponding decision table means that the transition relation defined by $res_{{\mathcal {A}}}$ in the reaction system ${\mathcal {A}}$ is, in a sense, consistent with the experimentally gathered data. This means that rules for transforming the identified set of entities in the perceived situation are the same as in the reaction system model.

Now, we are also ready to define perceived states of a given reaction systems ${\mathcal {A}}=(S,A)$ relative to decision systems $\mathbb {DT}(s)=(U,AT(s),d(s)),$ for $s\in S$.

##### **Definition 8**

Let $u\in U$ be a physical situation, let ${\mathcal {A}}=(S,A)$ be a reactions system, and let $\mathbb {DT}(s)=(U,AT(s),d(s))$ be a decision system corresponding to the entity *s*,  for $s\in S,$ with ${\mathbb{IS}}=(U,AT(s)).$ The *perceived state* of a reaction system ${\mathcal {A}}=(S,A)$ corresponding to $u\in U$ (relative to $\mathbb {DT}(s)$ for $s\in S$) is defined by

$$\begin{aligned} \underline{S}_u=\left\{ s\in S{:}\,u\in {\mathsf {LOW}}_{AT(s)}(C(s))\right\} . \end{aligned}$$

Using the rules discussed above one can predict the successor state of $\underline{S}_u$ in ${\mathcal {A}}$ as $res_{{\mathcal {A}}}(\underline{S}_u).$

Since the perception of entities in physical situations is only partial, instead of the perceived state $\underline{S}_u$ one can consider a pair of states $(\underline{S}_u, \overline{S}_u)$ defined by the lower and the upper approximations, where

$$\begin{aligned} \overline{S}_u=\left\{ s\in S{:}\,u\in {\mathsf {UPP}}_{AT(s)}(C(s))\right\} . \end{aligned}$$

Now, one can consider to have $\overline{S}_u$ as a model of the current state, or rather, due to uncertainty in the state identification, a family $\{\underline{S}_u \cup S^{\prime }{:}\,S^{\prime }\subseteq \overline{S}_u {\setminus } \underline{S}_u\}$ as a model of possible current states, and

$$\begin{aligned} \{res_{{\mathcal {A}}}(\underline{S}_u \cup S^{\prime }){:}\,S^{\prime }\subseteq \overline{S}_u {\setminus } \underline{S}_u\} \end{aligned}$$

as the set of possible successor states.

Hence, as the result of uncertainty in perceiving the physical reality, we obtain the nondeterminism in the prediction of the successor state.

For more details on relationships of rough sets and reaction systems readers are referred to Dutta et al. (2018), where, in particular, relationships of rough sets with exploration systems are discussed (Ehrenfeucht and Rozenberg 2014, 2015).

Using the proposed modeling, one can expect to obtain a set of such rules describing exact dependencies between approximated regions. Moreover, one may use more advanced methods of approximation such as Variable Precision Rough Set Model (Ziarko 1993).

Finally, a model of transition relation can be represented by a set of rules. These models may change when the accumulated data are changing (by adding new states, attributes or methods of aggregation). Hence, one may also look for learning methods for prediction how such sets of rules are changing (on the basis of the accumulated data and knowledge). This problem of evolving models of transition relations with time when conditions in the environment are changing is one of the important issue to be studied. Let us also note that the discussed sets of rules may be used for inducing concurrent models consistent with such sets of rules (see e.g., Pawlak 1992; Skowron and Suraj 1993, 1995; Suraj 2000).

The proposed model based on the rough set approach seems to be also suitable for modeling situations related to different contexts in which reactions are performed as well as for learning dependencies between different levels of hierarchical modeling (such as modeling on the level of biochemical reactions in cells and the level of cells concerning behavioral patterns of cells). Further studies are needed to clarify the usefulness of the proposed approach in modeling complex phenomena occurring in real-life applications. Another problem to which the proposed approach seems to be very suitable, concerns about the control of reaction systems. Also relations with other approaches, like aggregation of information systems into networks of information systems in the context of information flow approach (Barwise and Seligman 1997) and zoom structures (Ehrenfeucht and Rozenberg 2014) need to be further explored.

Let us now turn back to the discussion on IGrC.

### Rudiments of complex granules and interactive granular computing

Recently many researchers emphasize that models of computations should be based on the physical reality. This concerns in particular the process of learning from environment. More specifically, in Vapnik (1998) the need for considering the physical world as the basis for computations in the context of problems in applications is well expressed:

further study of this [learning] phenomenon requires analysis that goes beyond pure mathematical models. As does any branch of natural science, learning theory has two sides:

- The mathematical side that describes laws of generalization which are valid for all possible worlds and
- The physical side that describes laws which are valid for our specific world, the world where we have to solve our applied tasks.

[…] To be successful, learning machines must use structures on the set of functions that are appropriate for problems of our world. […] Constructing the physical part of the theory and unifying it with the mathematical part should be considered as one of the main goals of statistical learning theory. […] In spite of all results obtained, statistical learning theory is only in its infancy…

According to Vapnik (1998), there are many branches of the learning theory that have not yet been analyzed and that are important both for understanding the phenomenon of learning and for practical applications. Definitely, one of such area of the research should consider the necessity of linking the abstract world of mathematics with the physical world. This may be related to the grounding problem investigated in psychology (Anderson 2007; Harnad 1987, 1990; Jankowski 2017). In this paper we follow the approach based on complex granules (c-granules, for short) aiming to link these two worlds (see e.g., Jankowski 2017; Jankowski et al. 2014b, 2015; Skowron and Jankowski 2016a, b, c; Skowron et al. 2012a, 2016).

One of the main assumptions in interactive computations on c-granules is that the computations are based on physical objects. These physical objects, e.g., control tools for following some schemes for measurements and objects which are to be measured, are interacting among themselves. These activities take place in the physical world (i.e., *P* of Fig. 5). The results of these interactions are recognized (measured) by a given agent *ag* using so called measurable objects, i.e., objects whose states at a given moment of time *t* may be measured. The values of measurements are represented as values of attributes (e.g., real numbers) or degrees of satisfiability of some formulas. This pertains to the activity of abstract world (cf. Fig. 5). Using measurable objects the agent may indirectly recognize properties of other physical objects, which are not-directly measurable, in a given configuration provided *ag* has learned relevant interaction rules to predict changes of states of such objects on the basis of measurement performed on the measurable objects. Information about states of non-directly measurable objects to measurable objects is transmitted through interactions in the considered configuration.

**Fig. 4:**
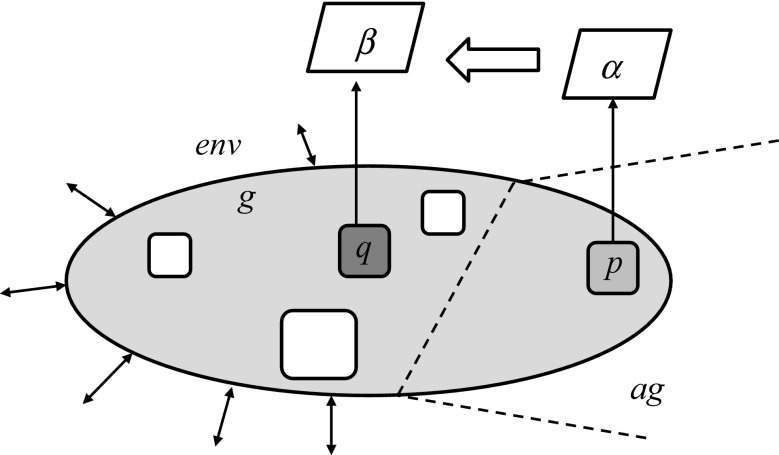
Learning interaction rule

In Fig. 4 we can see the gray part represents the c-granule *g* lying in the *ag*’s environment *env*. Over some time interval $[t-\varDelta ,t]$ based on interaction $Int_{g,t,\varDelta }(env,ag)$ between *env* and *ag*, a rule, say $\frac{\alpha ,\gamma }{\beta }$ is learned by *ag*. We can consider that the formula $\gamma$ represents the properties of the structure (e.g., mereology) of *g*, $\alpha$ describes property of the interaction process $Int_{g,t,\varDelta }(env,ag)$ in $[t-\varDelta ,t]$ at a measurable (by *ag*) part *p* of *g*, and $\beta$ describes expected property of the interaction process in $[t-\varDelta ,t]$ at a non-measurable (by *ag*) part *q* of *g* (where $t>\varDelta$).

**Fig. 5:**
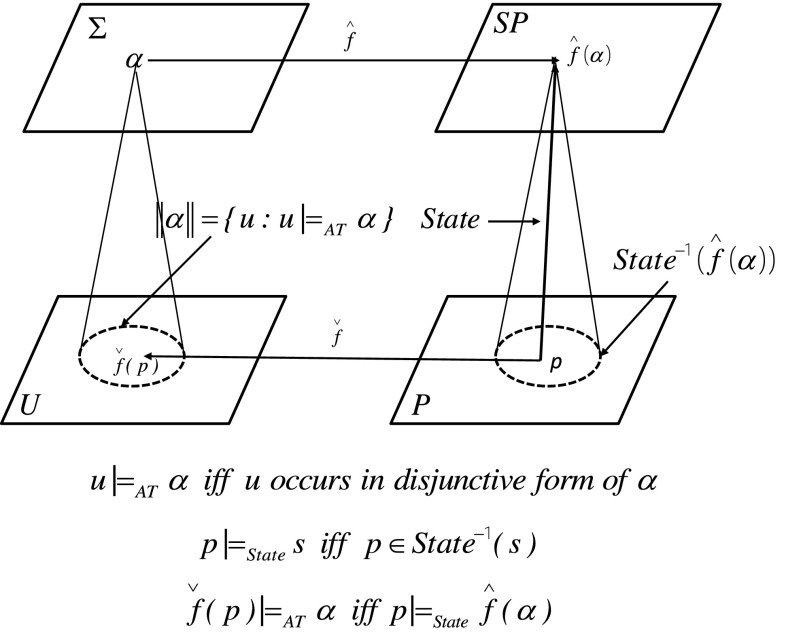
Infomorphism from the abstract world to the physical world

Using the information flow approach by Barwise and Seligman (1997), in particular using the definition of infomorphism, one can explain how the abstract part, related to measurable objects, is conjugated to physical objects (see Fig. 5). The abstract world is represented by a set of formulas $\varSigma$ [e.g., consisting of boolean combinations of descriptors over a given set of attributes *AT* (Pawlak and Skowron 2007)] and the set *U* of *AT*-signatures of objects, where *AT* is a set of attributes. The satisfiability relation $\models _{AT}$ is defined by $u\models _{AT} \alpha$ iff *u* occurs in one of the components of the disjunctive form of $\alpha$. The abstract world is defined by a classification $(U, \varSigma , \models _{AT})$ (Barwise and Seligman 1997). *P* denotes the set of physical objects, and *SP* is the set of states of physical objects. Moreover, $State{:}\,P\rightarrow SP$. The satisfiability relation for the physical world is defined by $p\models _{State} s$ iff $p\in State^{-1}(s)$^5^ for any $p\in P$ and $s\in SP$. The physical world is defined by a classification $(P, SP, \models _{State})$ (Barwise and Seligman 1997). A pair of functions $(\hat{f},\check{f})$ is an infomorphism from the abstract world to the physical world iff the condition at the bottom of the figure holds for all $p\in P$ and $\alpha \in \varSigma$ (Barwise and Seligman 1997).

The fundamental intuition behind the concept of a c-granule is the following:

Each of c-granule *g* has three distinguished architectural layers:

1. *Soft_suit* consists of configurations of hunks [i.e., physical objects treated as four-dimensional hunks of matter (Heller 1990)], called information granules, and it represents the properties of the environment of activity of *g* (including the properties of the present, past, and expected phenomena, as well as expected properties of interaction plans and/or the results of some interactions, potentially activated by *g*).
2. *Link_suit* consists of links (communication channels) which transmit the results of interactions among accessible fragments of the environment of activities of *g* and the results of interactions among different representations of properties in the soft_suit according to the weight (significance) of the current needs of *g*. These links may assign priorities to weights, which reflect the results of judgment by *g*.
3. *Hard_suit* consists of configurations of hunks accessible by links from the link_suit.

The hard_suits, link_suits, and soft_suits of more compound c-granules are formed by aggregating the relevant networks of already defined c-granules. The networks may satisfy some constraints, which can be interpreted as definitions of types of networks. The link_suits of such more compound granules are responsible for transmission of interactions between the hard_suits and soft_suits represented by the corresponding networks. The results and/or properties of transmitted interactions are recorded in the soft_suits.

In any c-granule, the interactions, which take place in the hard_suit, are transmitted through link_suit to the soft_suit, and these are recorded in the soft_suit. This is typical for sensory measurement. On the other hand, a c-granule may cause some interactions in its hard_suit by transmitting some interactions from the soft_suit through its link_suit. However, the c-granule may perceive the results (or properties) of such interactions, caused in the hard_suit, only by using the soft_suit. This is done on the basis of the transmitted results (or properties) of these caused in the hard_suit interactions in the hard_suit by transmitting them back through the link_suit to the soft_suit. These results (or properties) may be different from the predicted ones, which are a priori stored in soft_suit. This is typical for performing of actions initiated by c-granules.

C-granules are generated by an agent *ag* depending on the specific configurations of spatio–temporal portions of physical matter [called hunks (Heller 1990)] related to the *ag*. It should be noted that any typical active c-granule is a dynamically changing entity. It means that all components of c-granules (i.e., soft_suit, link_suit and hard_suit) are usually subject to continuous changes.

### Open information systems and dynamic networks of information systems in modeling of c-granules

It is worthwhile mentioning that the considered information systems (decision systems) should be considered not as closed objects; rather in the context of c-granules they interact with the environment. This means that these systems should be treated as open information systems. Moreover, developing methods for concept approximation, based on networks of information systems changing with time, is needed. One of the important departures, in such information systems is that instead of value sets of attributes relational structures over the value sets together with set of formulas interpreted over such structures (Skowron and Dutta 2017) are considered. This makes the process of modeling relevant granules in searching for relevant computational building blocks (patterns) for the complex vague concept approximations challenging. These concepts are used as guards for initiating actions performed by agents (Jankowski 2017; Skowron and Dutta 2017).

**Fig. 6:**
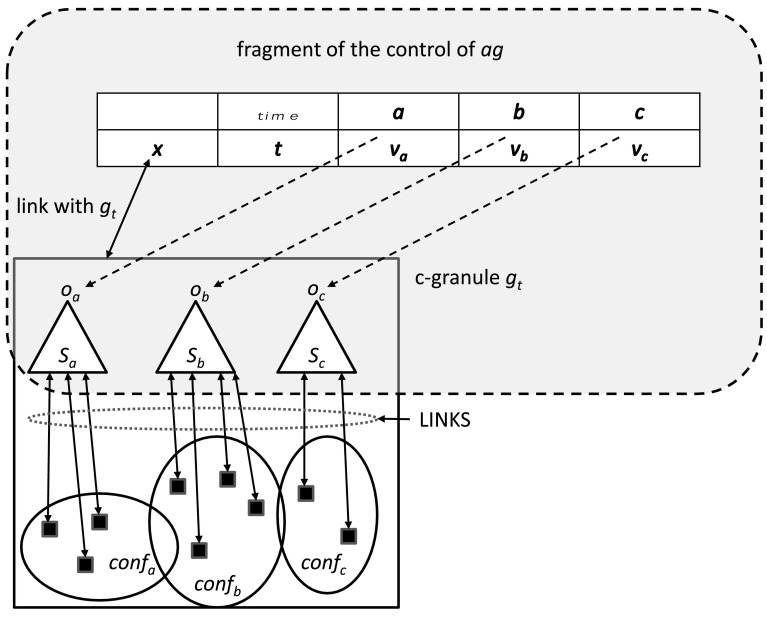
An illustrative fragment of the control of agent *ag* for acquiring values $v_a, v_b, v_c$ of attributes *a*, *b*, *c* using interactions of the control of *ag* with the c-granule $g_t$ created by *ag* at the local time *t* of *ag*; $g_t$—c-granule created (or updated) at time *t* by *ag* for computing values of attributes *a*, *b*, *c*; $v_a, v_b, v_c$—values representing states at time *t* of objects $o_a,o_b,o_c$ obtained by aggregation of information delivered by links; $conf_a, conf_b, conf_c$—configurations of physical objects in $g_t$ related to attributes *a*, *b*, *c*; *LINKS*—set of links for transmitting results of interactions in configurations $conf_a, conf_b, conf_c$ to the measurable objects $o_a, o_b, o_c$ of *ag*; link with $g_t$ is responsible for storing values $v_a,v_b,v_c$ of attributes *a*, *b*, *c* corresponding to the states of objects $o_a,o_b,o_c$ at time *t*; *x* is a symbolic representation of $g_t$ together with a pointer to its physical implementation

Let us illustrate a formal way of introducing a kind of adaptive information system. From general perspective, the ground for an adaptive information system is as follows. On the basis of interactions of an agent with the environment, using some control parameters, information systems (decision systems) are created. In particular, control parameters are used to perform some actions or plans on some distinguished physical objects for predicting different values of parameters about the physical objects. This process of controlling the schemes for obtaining values of attributes by fixing control parameters may be called as an *agent’s control*. In general, by fixing the control parameters, e.g., space-time location, position of sensors or/and actuators etc., the agent prepares the ground for obtaining an information system describing the properties of real physical objects. These real physical objects along with the set-up of the control tools (i.e., space-time-angle of sensors or cameras) generates a complex granule (c-granule, for short) (Jankowski 2017; Skowron and Jankowski 2016b). These c-granules, parts of c-granules, relationships among them, features of parts of the c-granules, and links of c-granules all together help to transmit the results of interactions with objects to the so called information tables (see Fig. 6). The complex c-granule lying in the reality represents the *physical world*, denoted as *P* in Fig. 5. On the other hand, the information tables basically represent the states of the measurable physical objects lying in the c-granules in terms of values of attributes; this is part of the *abstract world*, information about which is represented by some formulas (cf. $\varSigma$ in Fig. 5).

### Adaptive rough sets and adaptive reasoning

**Fig. 7:**
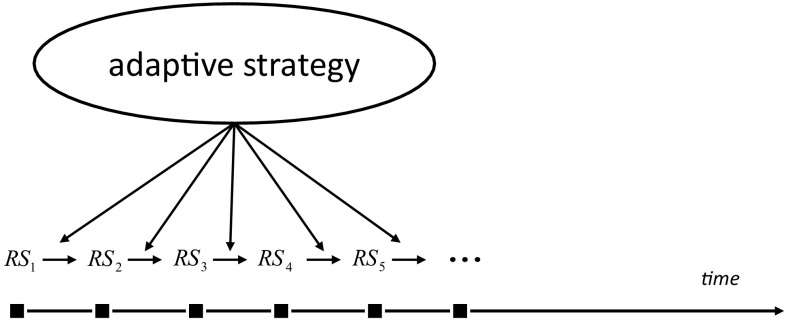
Adaptive rough sets

Following the already existing literature it is well known that vague concepts cannot be approximated with a satisfactory quality by *static* constructs such as induced membership/inclusion functions, or models that are derived from a sample. Understanding of vague concepts can only be realized in a process in which the induced models are adaptively matching the concepts in a dynamically changing environment. This conclusion seems to have important consequences for further development of rough set theory, in combination with fuzzy sets and other soft computing paradigms, towards adaptive approximate reasoning. For further details readers are referred to Skowron (2005) and Skowron and Swiniarski (2005). Due to different aspects of changes, an agent’s perception about a vague concept gets adapted with dynamically changing environment and time. Thus, we obtain a family of lower approximations, upper approximations and boundary regions of a considered vague concept in accordance with changing time (see Fig. 7).

From the above considerations it follows that for dealing with higher order vagueness one should consider all the above possibilities in the formal definition of rough sets. This also concerns the definition of information systems. In the following sequel we take an attempt to throw light on the issues regarding how to extend the present notion of information systems. We focus on the aspects that different perspectives of a concept may come due to viewing a set of objects with respect to different sets of attributes, or perceiving the same concept with respect to different sets of objects as well as attributes, or having change of perception with the appearance of new objects along the progress of time. Though we admit the phenomenon of higher order vagueness, we need to still fix the approximate understanding of a concept at some level, and for that we need to have different strategies for aggregating different perspectives of a vague concept. In order to aggregate information available at different information systems, a notion of interaction between information systems, mathematically which may be called *infomorphism* following (Barwise and Seligman 1997), will play an important role.

The control of an agent is responsible for predicting values of parameters necessary for constructing the relevant current information system. This prediction is performed on the basis of knowledge accumulated in the memory of control. The aim of the control of an agent is to satisfy the needs of the agent by controlling computations on c-granules. The algorithms, called classifiers (or regressors), for predicting the values of parameters are induced on the basis of information dynamically accumulated by the agent in the form of interactive information (decision) systems. These systems are dynamically changing with time due to interactions of the control with the environment. The process of inducing classifiers (or regressors) is often supported using hierarchical learning (e.g., Bazan 2008; Jankowski 2017; Skowron and Szczuka 2009).

Moreover, we would like to emphasize the necessity of developing adaptive strategies on the basis of the history/memory of control, which can guide how the information is gathered in such interactive systems, as well as how the structures of classifiers (regressors) are used in the past for predicting values of parameters. All these are inducing the high quality classifiers (regressors) for predicting values of the parameters for the current situation. The challenge is to develop methods for learning classifiers (regressors) for predicting adaptation of parameters based on what the agent already learnt about the perceived changes in situations and in the classifiers (regressors). The induced classifiers (regressors) can be treated as the temporary approximations of the decision functions (see Skowron et al. 2018; Skowron and Nguyen 2013).

The imprecise nature of a concept is often caused due to the unavailability of the information about all possible objects of the discourse. An agent at some point of time *t* may become able to gauge some part of the reality by accessing some objects, lying in the real world, and certain properties of them. Thus, at time *t* the agent only becomes able to describe the nature of the real world by a vague/imprecise concept. At some further point of time $t^{\prime }$ the agent may manage to access some more objects relevant to the concerned fragment of the reality, and learn about their properties. This helps the agent to have a better description of the vague concept, fitting to the reality. The following quote by Noë (2004) regarding having a vague perception about reality and thereby generating vague concepts, may be proper here.

Think of a blind person tap-tipping his or her way around a cluttered space, perceiving that space by touch, not all at once, but through time, by skillful probing and movement. This is or ought to be, our paradigm of what perceiving is.

Keeping this in mind, we outline a set-up for departing from the notion of information system (Orłowska and Pawlak 1981; Pawlak 1973a, b, 1981, 1991; Pawlak and Skowron 2007) to a notion of *Adaptive Information System* (AIS). In order to do so, below, we first present an intuitive background of the proposed formalism.

**Fig. 8:**
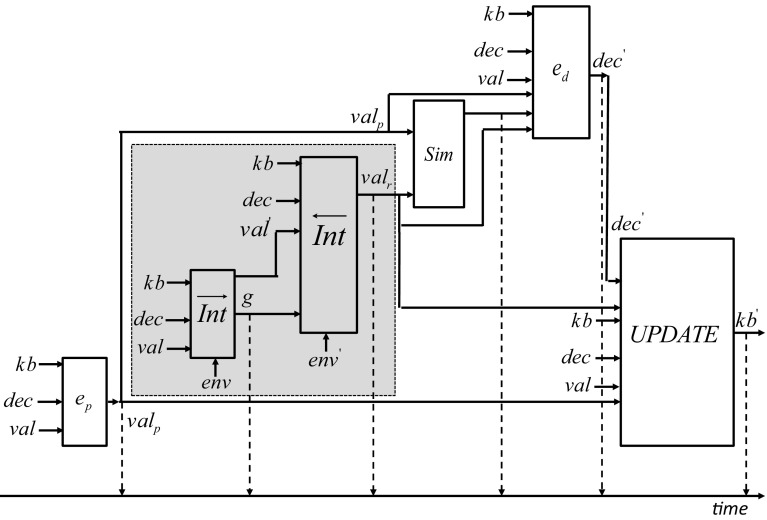
Basic cycle

In Fig. 8 we present an illustrative basic cycle of the control of agent *ag*.

First the value of function $e_p$ is computed on the basis of the agent’s knowledge base state *kb*, valuation of decision attributes *dec*, and valuation *val* of conditional attributes from *AT*. The predicted valuation $val_p$ of conditional attributes from *AT* is provided by $e_p$. The gray box in Fig. 8 illustrates the process of construction (by operation $\overrightarrow{Int}$) of c-granule g, in particular on the basis of the values of decision attributes (control parameters). It also shows in the c-granule *g*, using the link for transmission of interactions (operation $\overleftarrow{Int}$) from hard_suit to soft_suit, that the (real) evaluation $val_r$ of conditional attributes (which can be different from the predicted ones) is realized. This box is related to the Vapnik (1998) remark:

[…] further study of this [learning] phenomenon requires analysis that goes beyond pure mathematical models…

Next valuations $val_p, val_r$ (i.e., functions defined on conditional attributes with values in the value sets of these attributes) are compared by operation *Sim*,  and as a result a degree of similarity between $val_p$ and $val_r$ is obtained. This degree together with the valuations of conditional attributes $val_p, val_r$, valuation of control parameters *dec*, and the control state of knowledge base *kb*, are used to adapt the values of decision parameters (decision attributes) by operation $e_d$. As a result the revised valuation $dec^{\prime }$ of control parameters is obtained. The computed entities are next used to update the contents of knowledge base, and the new state $kb^{\prime }$ is obtained. This cycle illustrates an idea of adaptation of control parameters.

We would like to emphasize two basic problems related to the discussed adaptation.

Functions presented in Fig. 8 such as $e_p, e_r, Sim, UPDATE, e_d$ should be learned by the agent. They can be learned on the basis of partial information about these functions stored in the knowledge base. For each of these functions, such information has usually a form of a decision system. Hence, the agent should have strategies to learn from such partial information the models of the functions, making it possible to compute their values for new situations, which are not yet stored in the decision systems.

In the case of the gray area, the agent should be ready to learn the rules of interactions, allowing her/him to perceive if the c-granule *g* has been properly constructed, and predict the results of interactions (at least in typical situations) transmitted by *g*. These results are transformed into values of conditional attributes. Note that the obtained values depend also on the state of the environment *env* which can be changed in an unpredictable way. Hence, the conclusions obtained by using interaction rules may be treated only as hypotheses. The interaction rules are related to the above mentioned point view of Vapnik (1998) about necessity of the second component of learning consisting of:

The physical side that describes laws which are valid for our specific world, the world where we have to solve our applied tasks.

### Adaptive judgment in reasoning about interactive granular computations

Rough set theory has contributed to some extent to various kinds of deductive reasoning. Particularly, various kinds of logics based on the rough set approach have been investigated, rough set methodology has contributed essentially to modal logics, many-valued logics (especially different types of 3-valued logics), intuitionistic logics, paraconsistent logics and others [see e.g., references in book (Skowron and Suraj 2013) and in articles (Pawlak and Skowron 2007)].

There are numerous issues related to approximate reasoning under uncertainty including inductive reasoning, abduction, analogy based reasoning and common sense reasoning.

We would like to stress that still much more work should be done to develop approximate reasoning methods about complex vague concepts for making progress in development of IS or CAS. It is worthwhile to refer here to an extension of the citation, presented before, by Valiant (http://people.seas.harvard.edu/~valiant/researchinterests.htm):

A fundamental question for artificial intelligence is to characterize the computational building blocks that are necessary for cognition. A specific challenge is to build on the success of machine learning so as to cover broader issues in intelligence. […] This requires, in particular a reconciliation between two contradictory characteristics – the apparent logical nature of reasoning and the statistical nature of learning.

Here, two more views are also very relevant. The first one is by Professor Lotfi A. Zadeh, the founder of fuzzy sets and the computing with words paradigm (see Zadeh 1999 and also http://www.cs.berkeley.edu/~zadeh/presentations.html):

Manipulation of perceptions plays a key role in human recognition, decision and execution processes. As a methodology, computing with words provides a foundation for a computational theory of perceptions - a theory which may have an important bearing on how humans make- and machines might make - perception-based rational decisions in an environment of imprecision, uncertainty and partial truth. […] computing with words, or CW for short, is a methodology in which the objects of computation are words and propositions drawn from a natural language.

Another view is by Judea Pearl (the 2011 winner of the ACM Turing Award, “for fundamental contributions to artificial intelligence through the development of a calculus for probabilistic and causal reasoning”) (Pearl 2009):

Traditional statistics is strong in devising ways of describing data and inferring distributional parameters from sample. Causal inference requires two additional ingredients: a science-friendly language for articulating causal knowledge, and a mathematical machinery for processing that knowledge, combining it with data and drawing new causal conclusions about a phenomenon.

**Fig. 9:**
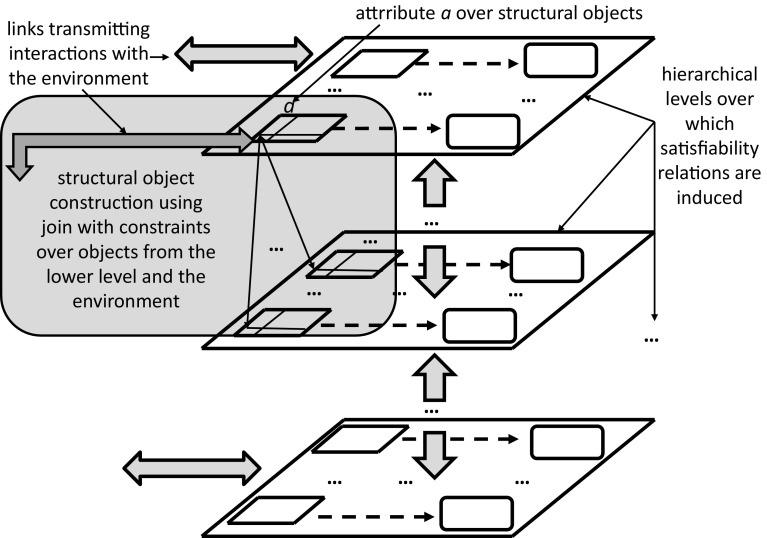
Interactive hierarchical structures (gray arrows show interactions between hierarchical levels and the environment, arrows at hierarchical levels point from information (decision) systems representing partial specifications of satisfiability relations to theories, induced from them, which consist of rule sets)

The question arises about the logic relevant for the above mentioned tasks. First let us observe that the satisfiability relations in the IRGrC framework can be treated as tools for constructing new information granules. If fact, for a given satisfiability relation, the semantics of formulas relative to this relation is defined. In this way the candidates for new relevant information granules are obtained. We would like to emphasize a very important feature that the relevant satisfiability relation for the considered problems is not given but it should be induced (discovered) on the basis of a partial information encoded in information (decision) systems. For real-life problems, it is often necessary to discover a hierarchy of satisfiability relations before we reach to the relevant target level. Information granules, constructed at different levels of this hierarchy, finally lead to relevant ones for approximation of complex vague concepts related to complex information granules expressed using natural language (see Fig. 9). The reasoning should also concern about how to derive relevant information granules for solutions of the target tasks, and that kind of reasoning is called adaptive judgment. Deduction, induction, abduction as well as analogy based reasoning all are involved in adaptive judgment. Among the different aspects, the following ones are a few which one needs to address as different subtasks in order to do reasoning with adaptive judgment.

- searching for relevant approximation spaces,
- discovery of new features,
- selection of relevant features,
- rule induction,
- discovery of inclusion measures,
- strategies for conflict resolution,
- adaptation of measures based on the minimum description length principle,
- reasoning about changes,
- perception (action and sensory) attributes selection,
- adaptation of quality measures over computations relative to agents,
- adaptation of object structures,
- discovery of relevant contexts,
- strategies for knowledge representation and interaction with knowledge bases,
- ontology acquisition and approximation,
- learning in dialogue of inclusion measures between information granules from different languages (e.g., the formal language of the system and the user natural language),
- strategies for adaptation of existing models,
- strategies for development and evolution of communication language among agents in distributed environments,
- strategies for risk management in distributed computational systems.

The discussed concepts such as interactive computation and adaptive judgment are some among the basic ingredients of the Wisdom Technology (WisTech) (Jankowski and Skowron 2007; Jankowski 2017). Let us mention here the WisTech meta-equation, which is *wisdom* = *interactions* + *adaptive judgment* + *knowledge*. In particular, extension of the rough set approach to interactive computations is one of the current challenges.

**Fig. 10:**
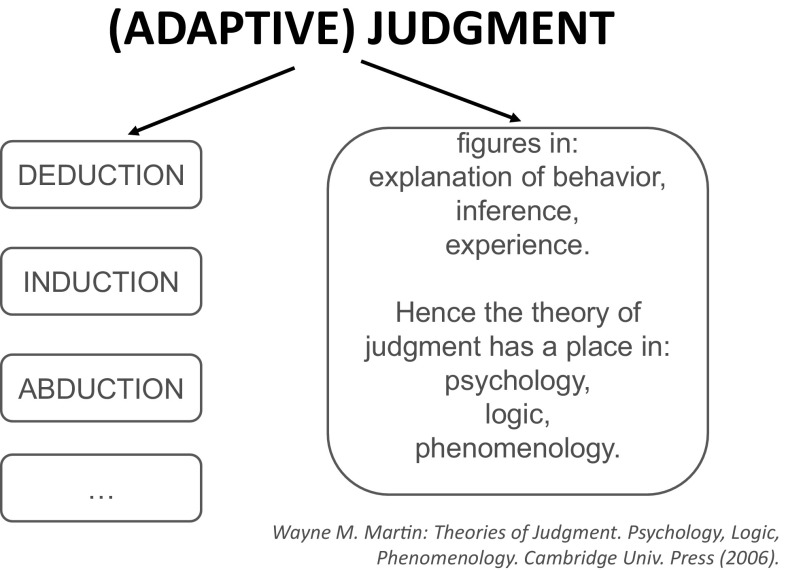
(Adaptive) judgment

Let us consider one more example of reasoning related to interactive computations on granules. In Fig. 8, one can see that iteration of the basic cycle leads to histories. A partial information about histories (e.g., in a form of time windows, or sequences of such windows) may be stored in knowledge base in the form of decision tables which can be used for inducing more advanced forms of adaptation of decision valuations (or function). The new decisions may depend not only on the current values of decisions but also on decisions contained in histories which are treated as objects in these more advanced decision systems. In this way modeling process of perception adjoined with actions mentioned before in the citation from Noë (2004) can be realized. One may observe that this process is related to hierarchical modeling and hierarchical learning, and it may be modeled using networks of information systems analogous to the Barwise–Seligman approach (Barwise and Seligman 1997). This process is also based on adaptive judgment (Jankowski 2017; Jankowski et al. 2014b, 2015; Skowron and Jankowski 2016a, b, c; Skowron et al. 2012a, 2016) with roots not only in logic, but also in psychology and phenomenology (Martin 2006) (see Fig. 10).

Definitely, the reasoning for adaptation should allow agents to base their reasoning on experience, what is the main concept of phenomenology. Hence, it is necessary to have good understanding of this concept for implementation in IS or CAS. This reasoning should allow agents to discover relevant patterns of behavior of other agents or objects, what is the subject of studies in psychology.

One can observe that this kind of reasoning is crucial for tasks, mentioned above by Vapnik (1998), in the sentence:

To be successful, learning machines must use structures on the set of functions that are appropriate for problems of our world.

Many advanced tasks, concerning complex systems may be classified as control tasks, performed by agents aiming at achieving the high quality computational trajectories of c-granules relative to the considered quality measures over the trajectories. Here, new challenges are to develop strategies to control, predict, and bound the behavior of the system. We propose to investigate these challenges using the IGrC framework.

Thanks to c-granules, it is possible to register both the results of sensory measures and their hierarchical aggregations, which are performed to discover new c-granules. The hierarchical c-granules discovered in this manner may ensure a deeper understanding of a perceived situation (see Bazan 2008). The statement above about the aggregation of c-granules (representing hierarchical aggregations of the results of sensory measures) refers to the main, according to Valiant,^6^ AI challenge, which is the characterization of “computational building blocks” for perception.

The key role in the proposed approach is played by the techniques of adaptive and interactive discovery of c-granules (through interactions with the environment) and their further use. It turns out that in order to perform computations on c-granules, *ecorithms*, as understood by Valiant (2013), should be used instead of classical algorithms. Apart from the analogy to Valiant’s ecorithms, the IGrC-based proposed algorithms display a number of other features, which correspond to the motivations of scientific research in other domains (e.g., learning systems, CAS, soft-computing, multi-agent systems, natural computations). The Wistech IGrC model is also related to the very foundations of AI, in particular, to the understanding of the essence of machine learning.

**Fig. 11:**
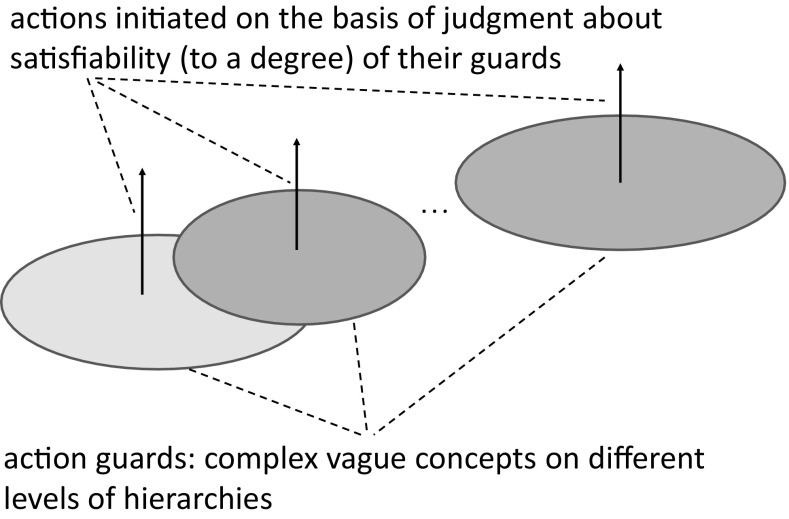
Complex games

In particular, in Complex Systems Engineering (CSE) (Jankowski 2017), the design and implementation of a complex project may be seen as the process of discovering, learning, processing (including communicating), and developing concepts (represented as c-granules), which are necessary to deal with a given project. The key to success in managing any complex project is a skillful approximation of complex vague concepts, represented by c-granules, and a skillful use of c-granules by those, who are in charge of a given project. Such approximations are responsible, e.g., for initiation of actions performed by agents (Jankowski 2017) (see Fig. 11) in complex-games.

**Fig. 12:**
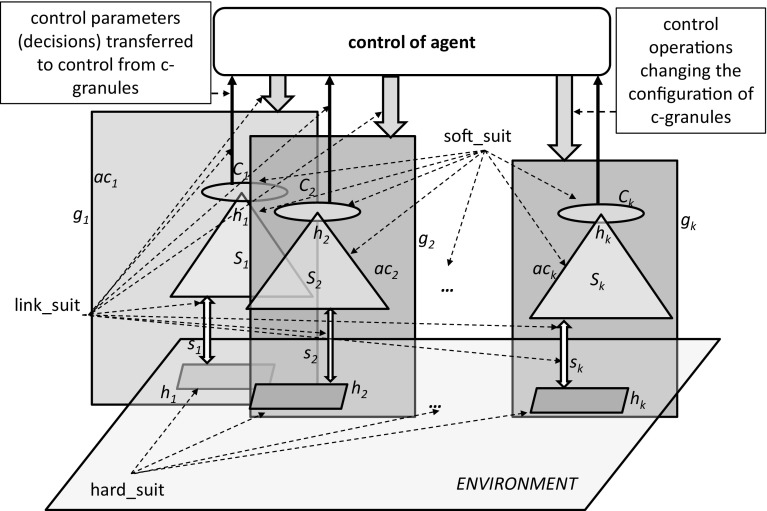
An example of configuration of c-granules and its interaction with the control of agent

Figure 12 illustrates reasoning in a process of changing the current configuration of c-granules on the basis of actions. This is realized on the basis of satisfiability of concepts responsible for triggering actions in the complex game. The actions are corresponding to predicted control parameters (decisions) transferred to the control. On this basis, control operations modify the current configuration of c-granules into a new one. The prediction of control parameters may also depend on the history of the past decisions and values of other attributes available to control. The control parameters are predicted on the basis of satisfiability of the complex vague concepts, denoted by $C_1, C_2, \ldots , C_k$ in the figure. These concepts are approximated by the aggregation schemata $S_1, S_2, \ldots , S_k$.

It is worthwhile mentioning that networks of information systems play an important role in configuration of c-granules presented in Fig. 12. They make it possible to gather data received from the environment, as a result of interaction of physical objects in the spatio–temporal scopes of c-granules, as well as to aggregate already constructed information systems into the new ones in hierarchical modeling (see aggregation schemata $S_1, S_2, \ldots , S_k$ in Fig. 12) toward searching for relevant computational building blocks (patterns). These computational building blocks are used for approximation of complex concepts, which help to predict decisions (control parameters). On the basis of that, the control performs actions ($ac_1,\ldots , ac_k$) resulting in a new configuration of c-granules and initiating interactions among them.

## Conclusions

In this paper, we have discussed some issues related to the development of rough sets over 35 years, together with some challenges for the rough set approach, especially in the environment where computations are progressing due to interactions between physical and abstract (information) granules, and where they can be controlled by performing actions activated on the basis of satisfiability (to a degree) of complex vague concepts, modeled by approximations.

## Footnotes

1 More general cases are considered, e.g., in articles Skowron and Stepaniuk (2010) and Skowron et al. (2012c).

2 $\left| {X}\right|$ denotes the cardinality of the set *X*.

3 https://en.wikipedia.org/wiki/Complex_adaptive_system.

4 The 2011 winner of the ACM Turing Award, the highest distinction in computer science, “for his fundamental contributions to the development of computational learning theory and to the broader theory of computer science”(http://people.seas.harvard.edu/~valiant/researchinterests.htm).

5 To simplify notation we write $State^{-1}(s)$ instead $State^{-1}(\{s\}).$

6 http://www.seas.harvard.edu/directory/valiant.
